# Outcome of Split Thickness Skin Grafting and Multiple Z-Plasties in Postburn Contractures of Groin and Perineum: A 15-Year Experience

**DOI:** 10.1155/2014/358526

**Published:** 2014-05-21

**Authors:** Wani Sajad, Raashid Hamid

**Affiliations:** ^1^Department of Plastic Surgery, SKIMS, Srinagar, Jammu and Kashmir 190011, India; ^2^Married Doctors Hostel, Block A, Room No. S2, SKIMS, Soura, Jammu and Kashmir 190011, India

## Abstract

*Background*. Groin and perineal burn contracture is a rare postburn sequel. Such postburn contractures causes distressing symptoms to the patients and in the management of these contractures, both functional and cosmetic appearance should be the primary concern. *Aims*. To study the outcome of surgical treatment (STSG and multiple Z-plasties) in postburn contractures of groin and perineum. * Material and Methods*. We conducted a study of 49 patients, with postburn groin and perineal contractures. Release of contracture with split thickness skin grafting (STSG) was done in 44 (89.79%) patients and release of contracture and closure by multiple Z-plasties was done in 5 (10.21%) patients. * Results*. Satisfactory functional and cosmetic outcome was seen in 44 (89.79%) patients. Minor secondary contractures of the graft were seen in 3 (6.81%) patients who were managed by physiotherapy and partial recurrence of the contracture in 4 (8.16%) patients required secondary surgery. * Conclusion*. We conclude that postburn contractures of the groin and perineum can be successfully treated with release of contracture followed by STSG with satisfactory functional and cosmetic results. Long term measures like regular physiotherapy, use of pressure garments, and messaging with emollient creams should not be neglected and should be instituted postoperatively to prevent secondary contractures of the graft and recurrence of the contracture.

## 1. Introduction

Perineum and groin constitute only 4–6% of total body surface area and are very important sites in the body anatomically and functionally. Isolated burns to the genitalia and perineum are not common [1–3]. These burns are of major concern to the patient as well as clinician [4]. Flame burns and scalds are common causes of perineal and genital burns [4–6]. Alcoholism is considered to be one of the leading predisposing factors in perineal and genital burns [7]. Child abuse is also a risk factor in perineal and genital burns [8]. “Chullah,” an earthen made stove in which wood is used as fuel in the rural areas of India, is important cause for the perineal burns [2]. Use of loose clothes during cooking, spilling of kerosene on the clothes from a burning stove, or explosion of such stoves are also associated with perineal and genital burns [9, 10].

Patient usually presents with difficulty in squatting, walking, sitting, urination, defecation, and sexual intercourse in married persons [2]. Since the contracture is not in a stabilized position, recurrent ulceration may occur and in exceptional cases squamous cell carcinoma (Marjolin's ulcer) may develop [11]. Various complications of perineal burn contracture like intestinal obstruction, anal stenosis with megarectum, and gluteal pouching with total effacement of the gluteal folds and hooding of the rectum have also been reported [12–14].

In the management of these contractures, both functional and cosmetic appearances should be the primary concern. Various surgical procedures have been used for the release of these contractures which range from simple release and grafting to a number of different flap procedures.

## 2. Material and Methods

A total of 49 patients were evaluated, analysed, and treated from May 1996 to May 2011. All patients were studied in detail. A detailed history was recorded from each patient, laying special emphasis on the time since initial burn, causative agent, percentage of body surface area involved, presenting symptoms, and any associated illness. A detailed general physical, systemic, and local examination was carried out on all patients.

All patients were subjected to surgery under general anaesthesia and the following operative procedures were performed:

1. release of contracture with split thickness skin grafting in 44 (89.79%) patients;
2. release of contracture and closure by multiple Z-plasties in 5 (10.21%) patients. Z-plasties used included 5-flap Z-plasties for 2 patients and simple Z for 3 patients.

First dressing was seen on third or fourth postoperative day and percentage of graft take/loss was noted. Complications, if any, were recorded. Indwelling urinary catheter drainage was instituted for 3 to 4 days postoperatively. Once the graft stabilized, patients were discharged and advised to wear compression garments. Regular physiotherapy and messaging with emollient creams were advised in all cases to avoid any recurrence of the contracture. Operated patients were followed and the results were analyzed according to the functional and cosmetic outcome; patient's satisfaction regarding the operative procedure and need for any secondary surgeries were recorded.

## 3. Results

Majority of the patients, 23 (46.94%), were in the age group of 16–30 years. The patient's ages ranged from 3 to 70 years with mean age of 18 years. 35 (71.43%) were females and 14 (28.57%) were males. 40 (81.63%) patients belonged to the rural areas, where most of the people use Kangri during the winter months to keep themselves warm. In 77.56% of the patients, postburn contractures of the groin and perineum were because of Kangri burn. Other less common causes were hot water (8.16%), open chulla (8.16%), and flame burn (6.12%). In our series the meantime of sustaining the burn injury was 7.1 years with maximum 16 years and minimum 2 years. In our series, 25 (51.02%) patients had isolated burns of groin and perineum and, in 24 (48.98%) patients, burns to groin and perineum were associated with burns to surrounding areas including external genitalia, lower abdomen, and upper thighs. Majority of the patients were brought with complaints of difficulty in squatting (97.95%) followed by limitation of movements of hip joints (93.87%) and impairment of gait. External genitalia were hidden under the web in 21 (42.85%) patients having bilateral groin contractures associated with contractures of the surrounding areas. Ulcerations of the contracture scar were seen in 3 (6.12%) patients, out of which 1 (2.04%) patient had developed squamous cell carcinoma (Marjolin's ulcer). Unilateral groin contractures were seen in 25 (51.02%) of the patients and bilateral groin contractures were seen in 21 (42.86%) patients. In 3 (6.12%) of the patients contracture was confined to perineum only.

In our series of 49 patients two types of operative procedures were performed (Table 1):

1. release of contracture with split thickness skin grafting;
2. release of contracture and closure by multiple Z-plasties.

In our series of 49 patients, 44 (89.79%) patients underwent release of contracture and the resulting raw area was covered with split thickness skin graft and tie-over dressing was applied to immobilize the graft. Minor secondary contractures of the graft were managed by physiotherapy and pressure garments.

21 (42.85%) patients having bilateral groin contractures underwent release of contracture with split thickness skin grafting. Most of these patients had webbing over symphysis pubis which had hidden the external genitalia. Such webs were also released. 20 (40.82%) patients underwent release of unilateral groin contracture with split thickness skin grafting and 5 (10.21%) patients underwent release of unilateral groin contracture and closure by multiple Z-plasties. 3 (6.12%) patients with perineal contracture only underwent release of contracture with split thickness skin grafting.

Postoperative hematoma formation under the graft was seen in 2 (4.54%) patients. Minimal patchy graft loss was seen in 4 (9.09%) patients, which was managed conservatively. Minor secondary contractures of the graft were seen in 3 (6.81%) patient. Partial recurrence of the contracture was seen in 4 (8.16%) patients who required secondary surgeries.

Postoperative outcome was measured by comparing with contralateral groin where involvement was unilateral and with subjective assessment from patient satisfaction regarding function, mobility, exposed genitalia, improvements in movements at hip joint including gait, improvement in squatting, and cosmetic improvement. Objective assessment was made by the surgeon regarding the measurements of landmarks of groin.

**Figure 1:**
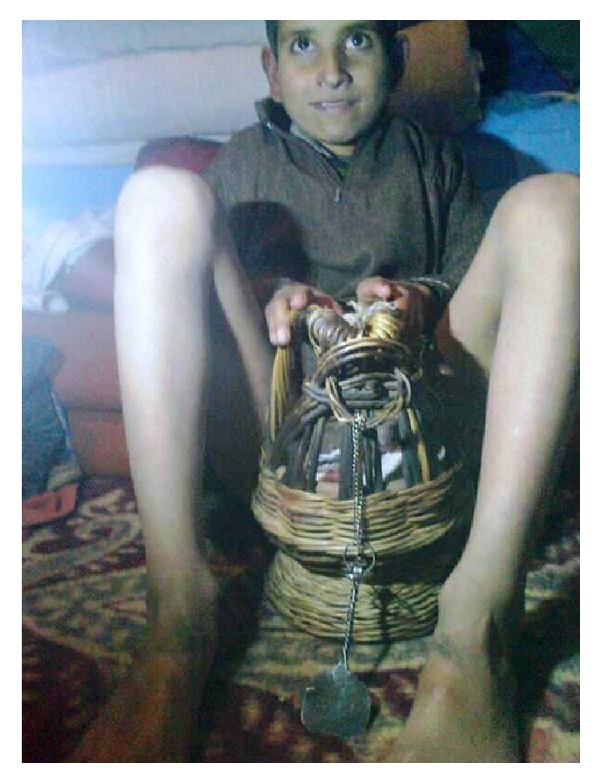
“Kangri” containing glowing charcoal, under a loose garment known as “Pheran” kept between medial aspect of thighs and lower abdomen in close proximity to groin.

**Figure 2:**
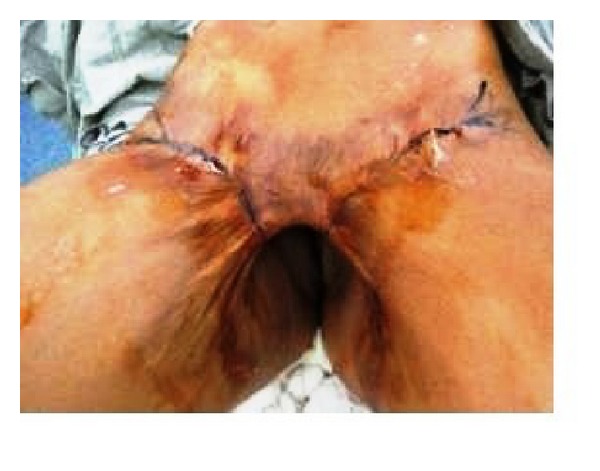
Bilateral postburn groin contracture with hidden genitalia under the web.

**Figure 3:**
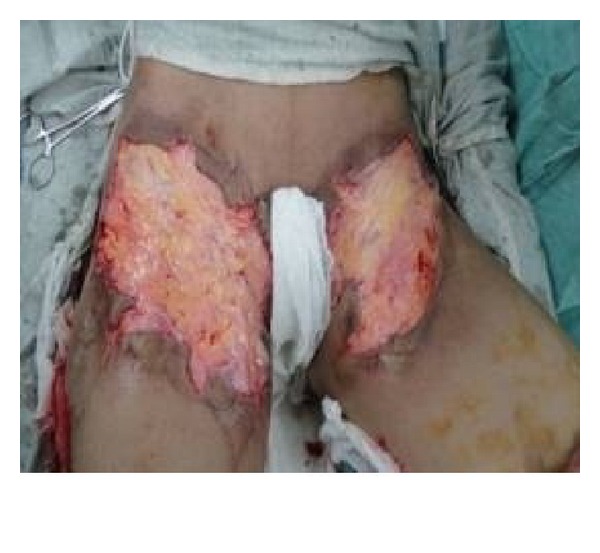
Raw area after release of contracture.

**Figure 4:**
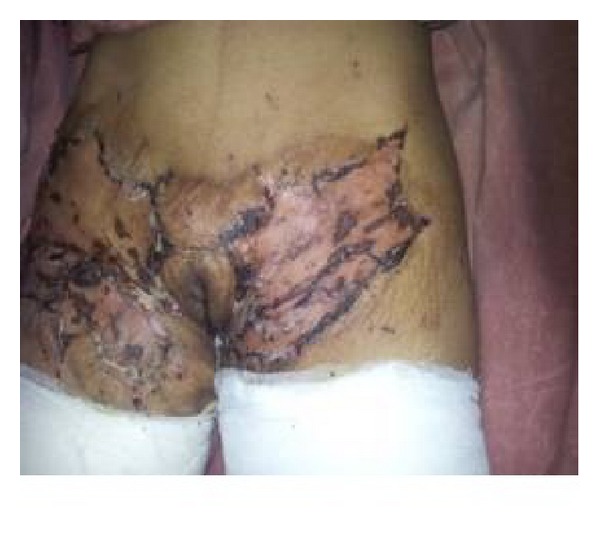
Split thickness skin graft in place in immediate postoperative view of patient shown in Figure 1.

**Figure 5:**
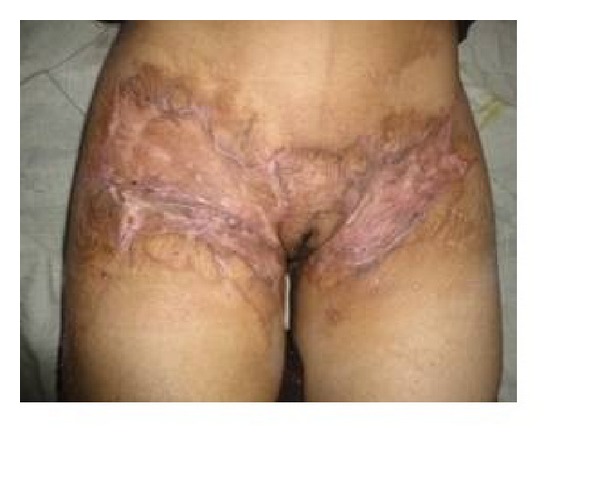
The same patient shown in Figure 1 in follow-up period. Genitalia exposed.

**Figure 6:**
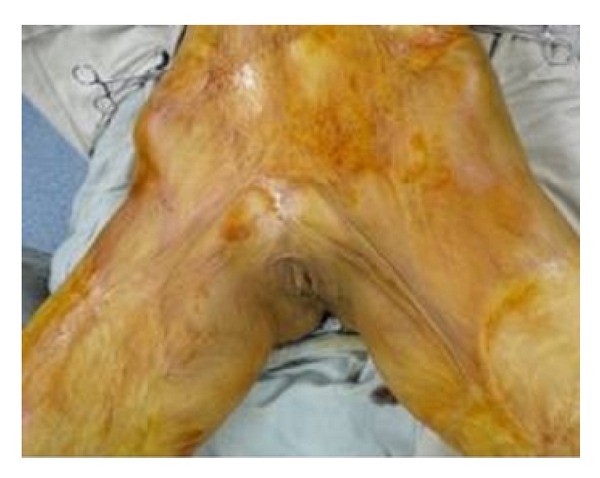
Bilateral postburn groin contracture.

**Figure 7:**
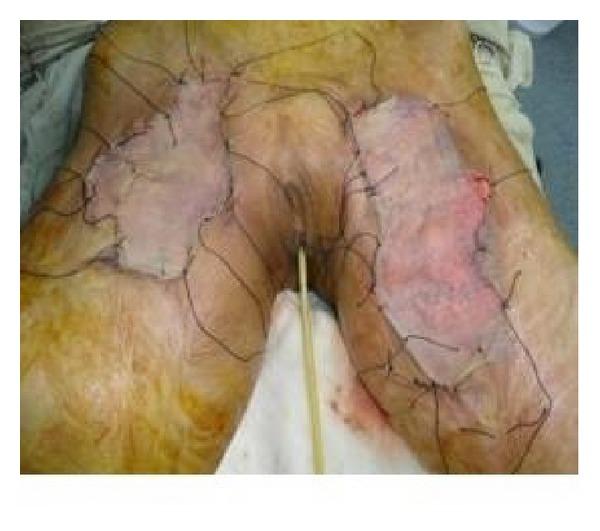
Split thickness skin grafting of raw area in Figure 5.

**Figure 8:**
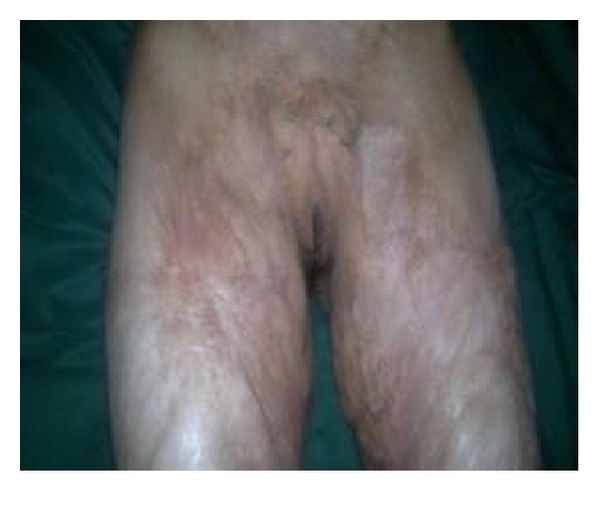
The same patient in Figure 5 on follow-up.

**Figure 9:**
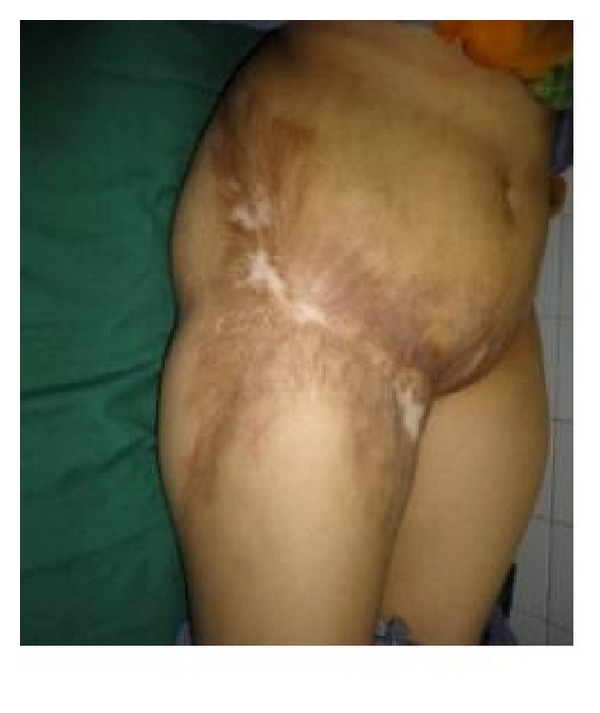
Right groin contracture with scarring.

**Figure 10:**
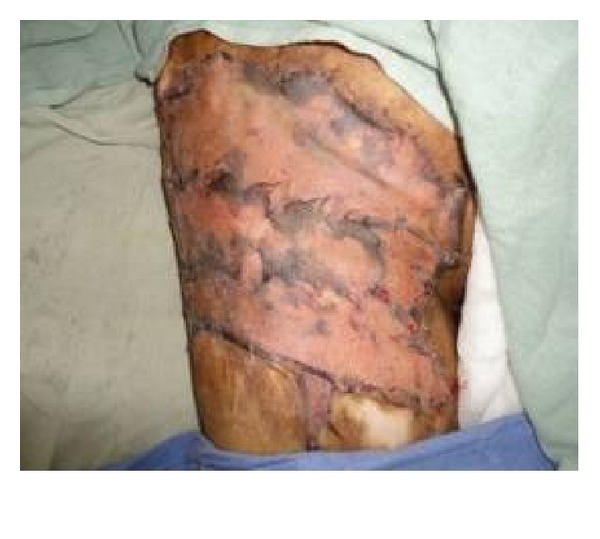
Immediate postoperative view after STSG of patient shown in Figure 8.

**Figure 11:**
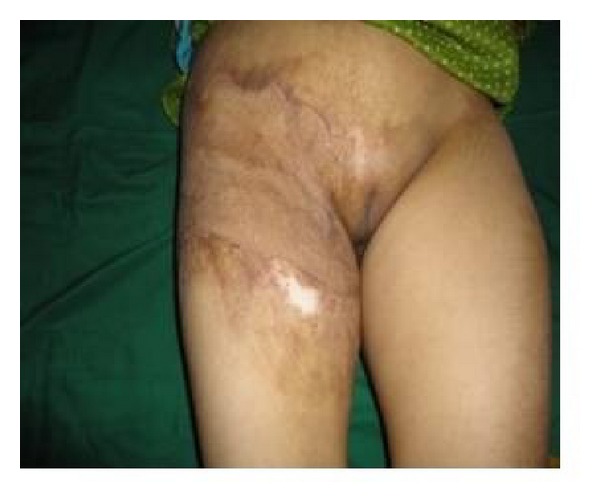
Follow-up view of the same patient shown in Figure 8.

Functional outcome was satisfactory in 44 (89.79%) patients; their squatting, walking, gait, and movements of the hip joints were improved and patients were able to perform all day to day activities of life and essential chores that require sitting or squatting position. In 4 (8.16%) patients functional outcome was not satisfactory. In these 4 patients partial recurrence of the contracture had occurred and required secondary surgeries. One patient died in follow-up due to road traffic accident. Cosmetic outcome was satisfactory in almost all except 4 (8.16%) patients, in which partial recurrence of the contracture had occurred (Figures 1, 2, 3, 4, 5, 6, 7, 8, 9, 10, and 11).

## 4. Discussion

Postburn contractures of groin and perineum are a rare burn sequel and such contractures are usually diagnosed late owing to the patient's negligence, ignorance, and shyness and delay can be extended until puberty and sometimes even later in females. The contracture band in the groin and across the symphysis pubis binds the thighs together, leading to functional problems like difficulty in squatting, walking, sitting, urination, defecation, and sexual function. Squatting, a common posture adopted in India for urination and defecation, was the main complaint in all our patients.

The mode of burn injury in our series was different from that reported by other authors. Sawhney [9] reported perineal burns sustained by spilling of kerosene on the clothes from a burning stove or due to explosion of such stoves. Bangma et al. [5] reported burns to the perineum and genitals due to scalds, explosion, open fire, or electricity. Balakrishnan et al. [7] reported perineal burns due to hot water, chemicals, and grease in males secondary to spouse abuse. Kumar et al. [6] reported scalds, flames, and electric burns as the most common contributors to burn injury. Michielsen et al. [4] reported burn injury to the genitalia and perineum mostly due to scalds, flames, and chemicals. Abdel-Razek [1] reported accidental chemical (sulphuric acid) burns to the genitalia. Thakur et al. [2] reported perineal burns caused by fire wood used in open “chullah.” In our series burns to the groin and perineum were mostly due to “Kangri” use (77.56%), open “chullah” (8.16%), hot water (8.16%), and flames (6.12%).

Because of the cold climate and poor economic conditions, majority of the people in Kashmir use “Kangri” to keep themselves warm during the winter months. “Kangri” is an earthen bowl containing glowing charcoal. It is used under a loose garment known as “Pheran” and is kept between medial aspect of thighs and lower abdomen in close proximity to groin and perineum. In majority of our patients (77.56%), accidental “Kangri” burn injury to groin and perineum was the mode of injury. Because these burns were not managed properly in acute phase, they had healed with the development of contractures of groin and perineum.

Perineal and genital burns are mostly part of large body surface injuries and isolated burns to perineum and genitalia are uncommon as reported by Abdel-Razek [1], Peck et al. [15], Bangma et al. [5], and Thakur et al. [2]. In patients with isolated burns to groin and perineum, “Kangri” was the sole causative agent responsible. This contradictory observation was due to the habitual use of “Kangri” in our state during the winter months.

Limitation of the movements of hip joints was seen in 46 (93.87%) patients. This limitation of movements of the hip joints had led to impairment of gait in 42 (85.71%) patients. Most of these patients had long standing contractures of groin. The findings are in agreement with the observations made by other investigators [2, 11, 16, 17].

As perineal burn contractures are not in a stabilized position, recurrent ulcerations may occur and, in exceptional cases, Marjolin's ulcer (squamous cell carcinoma) may develop [11]. Darzi and Chowdri [18] reported recurrent ulceration in 30 (41.66%) patients with postburn scar carcinoma. In our series we have seen recurrent ulcerations in 3 (6.12%) patients. Among these three patients, one patient, a 70-year-old male, had developed squamous cell carcinoma.

As reported by Sawhney [9], Rutan [19], and Thakur et al. [2], long term measures have to be instituted postoperatively to prevent skin graft contraction such as wearing tightly fitting undergarments. In our study also patients were advised to wear compression garments postoperatively. Regular physiotherapy and messaging with emollient creams were also advised in all cases to prevent any recurrence of the contracture. Postoperative follow-up ran smoothly with adequate healing. Squatting ability improved in 44 (89.79%) patients and they were able to perform essential chores that require squatting position. There were no donor site complications. Thus split thickness skin grafting was safe, less time consuming, and technically easy with good functional and cosmetic results. It can be done safely in patients having unilateral or bilateral postburn groin contractures and postburn perineal contractures of any severity.

Release of contracture and closure by multiple Z-plasties was done in 5 (10.21%) patients having minor unilateral postburn groin contractures in the form of linear bands. The functional and cosmetic results were satisfactory. Ye [20] found satisfactory results with local Z-plasty in 32 (82.05%) patients with postburn perianal scar contracture. Thus patients having minor postburn groin contractures, release of contracture and closure of wound by multiple Z-plasties can be done safely with shorter operative and recovery time and satisfactory functional results.

Besides graft and various types of Z-plasty used for release of groin contracture, the other clinical scenarios like extensive wounds after release of contracture, proximity to the anus and urethra, and wounds involving groin with exposure of femoral vessels after release of contracture may necessitate the use of flap cover. The sartorius muscle is transposed medially into groin for coverage of exposed femoral vessels. The gracilis muscle can be used to cover groin. The tenser fascia lata is useful for groin reconstruction. The rectus musculocutaneous flap based on its inferior pedicle provides a reliable flap cover. Free tissue transfer with anastomosis around femoral vessels remains a choice in difficult cases.

In our study satisfactory functional and cosmetic results were seen with split thickness skin grafts in patients having postburn contractures of groin and perineum. Our observations were consistent with the observations of other authors [1, 2, 4, 9, 11, 21].

## Figures and Tables

**Table 1:** 

Operative procedure	Number of patients	Percentage
Release of bilateral groin contracture with split thickness skin grafting	21	42.85
Release of unilateral groin contracture with split thickness skin grafting	20	40.82
Release of unilateral groin contracture and closure by multiple Z-plasties	5	10.21
Release of perineal contracture with split thickness skin grafting	3	6.12

Total	49	100.00
